# Biochar co-applied with silicon mitigates combined chromium and salinity stress in barley by upregulating antioxidant defense, hormonal balance, and nutrient homeostasis

**DOI:** 10.1039/d6ra01576j

**Published:** 2026-07-17

**Authors:** Imran Khan, Aqsa Andleeb Sial, Shazia Hanif, Muhammad Nawaz, Muhammad Umer Chattha, Ayesha Mustafa, Muhammad Bilal Chattha, Leeza Mujahid, Yasser S. Mostafa, Saad A. Alamri

**Affiliations:** a Department of Agronomy, University of Agriculture Faisalabad 38040 Pakistan drumer@uaf.edu.pk bilal1409@yahoo.com; b Department of Botany, University of Agriculture Faisalabad 38040 Pakistan; c Department of Agricultural Engineering, Khwaja Fareed University of Engineering and Information Technology Rahim Yar Khan Pakistan; d Department of Agronomy, University of the Punjab Lahore 54590 Pakistan; e Department of Biology, College of Science, King Khalid University Abha P.O. Box 9004 Saudi Arabia

## Abstract

Barley was grown for 150 days under controlled greenhouse conditions in pots containing 8 kg of soil. This study aimed to investigate the effects of co-applied biochar (BC) and silicon (Si) in mitigating combined chromium (Cr) and salinity stress (SS). Biochar was produced from sugarcane husks pyrolyzed at 500 °C and applied at 2% (w/w). Silicon (as sodium silicate) was applied at 150 mg kg^−1^. Chromium was added as K_2_Cr_2_O_7_ at 40 mg kg^−1^, and salinity stress was imposed at 12 dS m^−1^. The treatments included: control; Cr alone (40 mg kg^−1^); SS alone (12 dS m^−1^); combined Cr + SS; Cr + Si; Cr + BC; SS + Si; SS + BC; Cr + SS + Si; Cr + SS + BC; and Cr + SS + Si + BC. The results showed that combined Cr and SS reduced barley yield by 32.83% compared to the control. This reduction was associated with decreased chlorophyll contents (by 72.03–103.17%), leaf water content (by 75.55%), indole acetic acid (IAA; by 2.55-fold), gibberellin (GA; by 1.84-fold), soluble proteins (SP; by 2.14-fold), and soil nutrient availability (65.01%; average) and increased hydrogen peroxide (H_2_O_2_), malondialdehyde (MDA), abscisic acid (ABA; by 1.63-fold), Cr and Na accumulation in plant organs, and soil Cr availability. Co-application of BC and Si significantly reversed these negative effects and enhanced barley growth and yield. This improvement was achieved through increased antioxidant activity, higher chlorophyll content (22.22–35.59%), leaf water content (35.55%), IAA (46.64%), GA (42.17%), SP (45.87%), and soil nutrient availability; enhanced nutrient accumulation in plants; and reduced MDA (77.57%), H_2_O_2_ (72.40%), ABA (58.28%), soil Cr availability, and subsequent Cr accumulation in barley tissues. Therefore, co-application of BC and Si effectively mitigates combined Cr and SS in barley by improving plant physiology and soil nutrient availability while reducing Cr and Na uptake. These findings provide a foundation for developing strategies to counter the dual challenges of salinity stress and heavy metal contamination.

## Abstract 2

Barley was grown for 150 days under controlled greenhouse conditions in pots containing 8 kg of soil.

## Graphical abstract

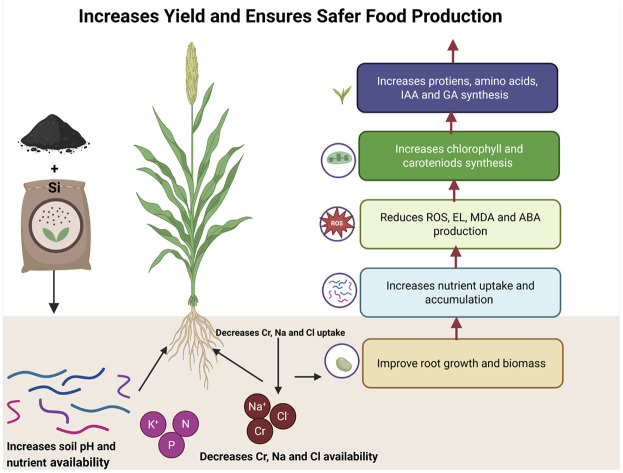

## Introduction

Soil degradation driven by heavy metal contamination has emerged as a critical threat to global food security. Heavy metals (HMs) are persistent, non-biodegradable contaminants that enter agricultural soils through industrial effluents, mining activities, electroplating, tannery waste, and sewage sludge disposal.^1^ Chromium (Cr) is most widely distributed and hazardous pollutants, particularly in irrigated agro-ecosystems of developing countries.^2^ It exists predominantly in two oxidative forms, Cr(iii) and Cr(vi), with Cr(vi) being highly mobile, soluble, and toxic.^3^ Its presence in soil not only threatens crop production and soil biological integrity but also contaminates the food chain, representing a significant public health concern.^4^ Chromium toxicity disrupts seed germination, inhibits radicle and plumule elongation, and limits cell division and mitotic activity.^5^ Physiologically, Cr interferes with photosynthetic machinery, reducing chlorophyll synthesis, impairing chloroplast ultrastructure, and inhibiting Rubisco enzyme activity.^6^ Moreover, it disturbs nutrient uptake and causes nutrient deficiency, thereby decreasing plant growth.^7,8^ A major biochemical consequence of Cr exposure is the excessive production of reactive oxygen species (ROS), which induces lipid peroxidation, membrane injury, protein oxidation, and DNA damage.^5^ Plants activate antioxidant activities to counter Cr toxicity; however, above certain thresholds, antioxidant capacity becomes insufficient, resulting in significant reduction in biomass productivity and physiological efficiency.^9^

Soil salinity is another global abiotic stress, affecting more than 6% of global land area (approximately 800 million hectares).^10,11^ Salinity stress causes water deficiency, inhibits photosynthesis, and increases ROS production, leading to cellular damage.^12–14^ It also decreases leaf area, root growth, electron transport, and photosystem-II efficiency, thereby reducing growth and biomass accumulation.^15,16^ Salinity stress also decreases stomatal conductance, transpiration rate, and photosynthetic rate ultimately reducing biomass production.^17^ In addition, salinity stress disturbs nutrient uptake by increasing sodium accumulation (by 222%) and decreasing potassium (by 17–33%), calcium (by 7.21%), magnesium (by 35.46%), and sulfur (by 45–56%) accumulation across different growth stages.^18^ Furthermore, salinity alters hormonal homeostasis, increasing abscisic acid (ABA; 2.6-fold) while decreasing gibberellins and cytokinins, which disrupts plant functioning and causes growth losses.^19^

Many regions globally faceing the dual challenges of salinity and HMs co-contamination.^20,21^ The co-occurrence of salts and HMs generally causes more severe negative impacts than either stress alone. A study on barley showed that co-exposure to salinity and Cr caused a more severe reduction in mineral concentration and accumulation (iron and manganese reduced by 51.70% and 39.69%, respectively).^22^ Similarly, combined application of 50 µM Cd and 100 mM NaCl increased Cd and Na accumulation by 4-folds in maize and caused a reduction of >80% in biomass yield by decreasing photosynthetic efficiency and increasing ROS production (by 30.01–52.73%) and membrane damage.^23^ Another study found that salinity and Cd together caused a considerable reduction in biomass and growth by increasing ROS production (by 38%) and membrane damage, while decreasing photosynthetic pigments and leaf gas exchange traits.^24^

Biochar (BC) is a promising strategy to mitigate HMs toxicity and improve the health of salt-affected soils.^5^ It has excellent functional groups and a large surface area, which immobilize HMs and reduce their accumulation in food crops.^25^ Biochar favors plant functioning and reshapes soil microbial communities, enhancing microbial network stability and thus alleviating SS.^26,27^ Biochar reduces HMs toxicity through adsorption, while its larger surface also facilitates the absorption of Na and heavy ions, thus reducing their uptake and accumulation by plants.^28^ Biochar application to Cr-polluted soils considerably decreases Cr availability to plants.^29^ Moreover, it lowers Cr pollution by reducing Cr availability, increasing soil pH, and improving plant functioning.^30^ Recently, the application of essential nutrients has shown tremendous potential to enhance crop productivity under stress conditions. Silicon (Si) is widely recognized as an important nutrient for enhancing salinity and HMs tolerance. Silicon application strengthens cell walls, boosts antioxidant activity, and improves photosynthetic efficiency, contributing to better growth under Cr stress.^31^ Silicon also improves chlorophyll pigments (by 55.54–61.35%), reduces MDA (by 15.60%) and ROS production (by 30.47%), and contributes to better growth.^32^ Under salt stress, Si maintains osmotic balance and chlorophyll synthesis, and increases the thickness of the Casparian strip, thus limiting Na movement from roots to aerial parts.^33,34^ Moreover, Si reduces HMs uptake and enhances nutrient uptake, contributing to the alleviation of combined salinity and HMs toxicity by lowering membrane damage and ROS production, while increasing antioxidant activities and modulating hormonal balance.^35^

Barley (*Hordeum vulgare* L.) is an important crop grown globally for feed, food, and malt production, with an annual production of approximately 140 million tons.^36^ Although it is moderately salt-tolerant (tolerating up to 8–10 dS m^−1^), its growth and productivity significantly decrease under combined salinity and HMs stress.^37^ Therefore, it is critical to identify effective strategies to reduce the combined toxicity of Cr and salinity on plants. The individual roles of salinity and Cr stress are well documented in the literature, however, the combined effects of Cr and salinity on plant physiology, biochemistry, and soil health remain poorly understood. Furthermore, Si and BC individually have shown appreciable results in mitigating salinity and Cr toxicity. Critically, the synergistic effect of co-applied BC and Si to mitigate combined salinity and Cr toxicity has not yet been studied. Therefore, the novelty of this study lies in the systematic assessment of BC + Si co-application as a dual-strategy intervention to simultaneously reduce Cr bioavailability, alleviate salinity stress, and improve barley growth under multi-contaminated conditions. This integrated approach provides a targeted, previously unexplored solution for reclaiming soils affected by both heavy metals and salinity. We hypothesized that co-applied BC and Si can more effectively mitigate combined Cr and SS toxicity than either amendment alone by improving plant physiological functioning, enhancing soil quality, and reducing Cr and Na accumulation. Therefore, this study aims to achieve the following objectives: (1) to quantify the individual and combined phytotoxic effects of Cr and SS on barley growth, physiology, and biochemistry; (2) to compare the stress-alleviating efficacy of BC + Si, testing the hypothesis that co-application produces synergistic protection; and (3) to elucidate the impacts of BC + Si on Cr/Na uptake, ionic homeostasis, antioxidant activity, and hormonal balance. The present findings address a critical gap in understanding how combined BC and Si can remediate salinity- and Cr-polluted soils. These findings will help develop cost-effective and sustainable soil management strategies to ensure safer crop production and food security.

## Materials and methods

### Experimental site, and soil collection

The experiment was conducted at the University of Agriculture Faisalabad, Pakistan, from November 2024 to March 2025. Soil was collected from the top 0–20 cm layer, sieved, and all debris was removed. The soil was loamy with an alkaline pH (7.8), electrical conductivity of 1.47 dS m^−1^, total nitrogen of 0.31 g kg^−1^, and available phosphorus and potassium contents of 12.58 and 163 mg kg^−1^, respectively.

### Treatments and experimental design

The experiment was laid out using a completely randomized design (CRD) with three replications. The treatments were as follows: T1: control; T2: Cr stress (40 mg kg^−1^); T3: salinity stress (12 dS m^−1^); T4: Cr stress (40 mg kg^−1^) + salinity stress (12 dS m^−1^); T5: Cr stress + Si (150 mg kg^−1^); T6: Cr stress + BC (2%); T7: salinity stress + Si; T8: salinity stress + BC; T9: Cr stress + salinity stress + Si; T10: Cr stress + salinity stress + BC; T11: Cr stress + salinity stress + Si + BC. Pots were filled with 8 kg of soil. Salinity stress was developed by dissolving NaCl in water and mixing it into the soil to achieve the desired EC level of 12 dS m^−1^ using the following formula:
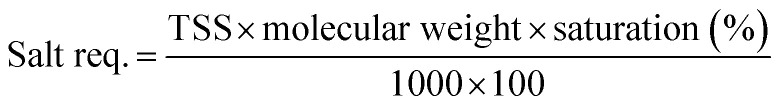


Total soluble salts (TSS) were calculated by multiplying the EC difference (required soil EC – starting soil EC) by 10 and the molecular weight of NaCl (58.5). Chromium stress was imposed by treating the soil with potassium dichromate (K_2_Cr_2_O_7_). Thereafter, the pots were maintained at 70% field capacity and placed in dark conditions for two months. After this period, BC and Si were mixed into the soil and allowed to stabilize for two weeks. Ten barley seeds were then sown in each pot. Silicon was applied in the form of potassium silicate (K_2_SiO_3_). Biochar was prepared from sugarcane residues through slow pyrolysis at 500 °C for 3 hours under limited oxygen supply. Before use, the BC was sieved (2 mm), and its properties were measured; details are provided in the Results section. The pH of the biochar was measured with a pH meter, while its surface morphology was studied using scanning electron microscopy (SEM). The presence of functional groups and nutrient concentrations in the biochar were measured using Fourier-transform infrared spectroscopy (FTIR) and energy-dispersive X-ray spectroscopy (EDX). After germination, five uniform seedlings were kept in each pot, and the remaining seedlings were removed. The pots were regularly irrigated, and all other practices were kept constant. The Sultan-17 barley cultivar was used in this study and it was obtained from the Ayub Agriculture Research Institute, Faisalabad. The biochar application rate (2%) was chosen based on previous literature showing its effectiveness in mitigating Cr and salinity stress.^38,39^ The silicon rate was similarly selected based on previous studies showing that this rate effectively alleviates abiotic stress toxicity in cereals. The Cr concentration and salinity level were chosen to simulate co-contamination. These levels were selected based on our previous studies showing that they reduce plant growth without causing complete mortality. Analytical grade reagents were used for analysis, with certified reference standards for calibration (*R*^2^ ≥ 0.999). Sterilized instruments and laboratory equipment were used throughout. All samples were analyzed in triplicate to obtain reliable results. Furthermore, each instrument was calibrated before every run. Calibration curves for Cr, Na, K, and Ca exhibited strong linearity (*R*^2^ > 0.998). Precision, expressed as the relative standard deviation of triplicate measurements, was <5%. Accuracy was verified through spike recovery tests (92–106%).

### Determination of leaf water status and photosynthetic pigments

Four weeks after sowing, fresh barley leaves were collected to assess relative water content (RWC). Leaves were immediately weighed after harvesting (fresh weight, FW), then hydrated in distilled water for 24 hours to obtain turgid weight (TW), and finally oven-dried at 70 °C to obtain dry weight (DW). RWC was calculated according to the method of Wasaya.^40^ Leaf photosynthetic pigments were determined following Lichtenthaler.^41^ Fresh leaf tissue (0.5 g) was homogenized using a pestle and mortar in 5 mL of 80% (v/v) methanol and incubated in the dark for 24 hours. The extract was centrifuged at 10 000 rpm for 10 minutes, and the supernatant was filtered. Absorbance of the clear extract was measured using a UV-visible spectrophotometer at 663 nm (chlorophyll a), 645 nm (chlorophyll b), and 480 nm (carotenoids).

### Determination of osmolytes and hormones

Total soluble protein (TSP) was determined following the method of Bradford.^42^ Leaf samples were homogenized in 5 mL of potassium phosphate buffer (PPB) and then centrifuged. The extract was mixed with 3 mL of Bradford reagent, and after 15 minutes, absorbance was read at 590 nm. Total free amino acids (TFAA) were estimated according to Hamilton and Van Slyke.^43^ An aliquot of 1 mL of crude extract was reacted with 1 mL of 2% ninhydrin and 1 mL of 10% pyridine, followed by incubation at 90 °C for 30 minutes. After cooling to room temperature, absorbance was recorded at 570 nm. For the measurement of ABA and IAA, 0.5 g of leaves was ground in 80% methanol containing 40 mg of butylated hydroxytoluene. The extract was then kept at 4 °C for 48 hours. Subsequently, the samples were homogenized for 15 minutes and then passed through C18 Sep-Pak cartridges using 10 mL of ether and ethanol. The resulting mixture was combined with 1% gelatin (pH 7.5) and 0.1% Tween-20 in phosphate-buffered saline. ABA and IAA contents were then measured according to the method of Weiler.^44^ For estimating GA concentration, 0.1 g of leaf slices was homogenized in 3 mL of 96% ethanol, and the absorbance was measured at 254 nm. GA concentration was calculated using a linear regression model according to Berrios.^45^

### Determination of oxidative markers and antioxidants

Membrane stability was assessed by measuring electrolyte leakage following the method described by Blum and Ebercon.^46^ Fresh leaf samples (0.5 g) were cut into small segments and immersed in a known volume of distilled water. The samples were incubated at room temperature for 30 min, after which the initial electrical conductivity (EC_1_) was measured. Subsequently, the samples were heated in a water bath at 90 °C for 30 min to release total electrolytes. After cooling to room temperature, the final electrical conductivity (EC_2_) was measured. Electrolyte leakage was calculated using the formula: electrolyte leakage (%) = EC1/EC2 × 100. To measure hydrogen peroxide (H_2_O_2_), leaves were subjected to homogenization by using 5 mL of trichloroacetic acid (TCA: 5%). Then, the mixture was centrifuged (10 000 rpm) for 20 minutes, and the supernatant was collected and later H_2_O_2_ was measured by following the methods of Velikova.^47^ For MDA, leaves were sliced into and 0.5 g samples were homongenized in TCA solution as mentioned above. Then, this mixture was centrifuged for 15 minutes and then subjected to heating (100 °C) for 30 minutes. Later, absorbance was noted at 532 nm.^48^

Antioxidant enzyme activities were determined using fresh leaf tissue. One gram of leaf material was homogenized in 10 mL of 50 mM potassium phosphate buffer (PPB, pH 7) under chilled conditions. The homogenate was centrifuged at 14 000 rpm for 30 minutes at 4 °C, and the resulting supernatant was used for enzyme assays. For catalase (CAT) activity, the reaction mixture consisted of 0.1 mL of enzyme extract, 0.1 mL of H_2_O_2_, and 2.5 mL of phosphate buffer. The decrease in absorbance was recorded at 240 nm following Chance and Maehly.^49^ Peroxidase (POD) activity was determined by mixing 100 µL of enzyme extract, 100 µL of 180 mM H_2_O_2_, 100 µL of 180 mM guaiacol, and 700 µL of 50 mM phosphate buffer. The increase in absorbance was measured at 470 nm. Ascorbate peroxidase (APX) activity was measured according to Asada and Takahashi.^50^ The reaction mixture contained 100 µL of enzyme extract, 100 µL of ascorbic acid, 6.1 mM H_2_O_2_, and 700 µL of phosphate buffer. Absorbance was recorded at 290 nm. Superoxide dismutase (SOD) activity was measured by reading absorbance at 560 nm according to the instructions of Zhang.^51^

### Measurement of chromium and nutrients accumulation and partitioning into plant tissues

Plant samples were powdered and digested in a mixture of HNO_3_ and HClO_4_ (2 : 1) according to Jones and Case.^52^ After complete digestion, Cr concentration in different plant parts was determined using atomic absorption spectrophotometry (AAS; PerkinElmer Analyst™ 800). Chromium content was calculated using the formula: Cr concentration = (AAS reading × dilution factor)/dry weight of sample. For nutrient concentration, root and shoot samples were digested using the same method. Nitrogen (N) and phosphorus (P) were estimated using the Kjeldahl and spectrophotometer methods, respectively. Sodium (Na), and potassium (K) were measured were measured using a flame photometer. Calcium (Ca), and magnesium (Mg) were measured with an atomic absorption spectrophotometer.

### Measurement of soil properties and growth and yield attributes

Soil samples were digested in a 2 : 1 mixture of nitric acid (HNO_3_) and perchloric acid (HClO_4_). After digestion, the samples were diluted, and soil Cr content was measured using AAS. Soil pH was measured in a 1 : 5 soil-to-water solution using a pH meter. TN was estimated using the Kjeldahl method, while AP and AK were estimated using spectrophotometer and flame photometer methods, respectively. Soil organic carbon (SOC) content was estimated using the potassium dichromate heating method with concentrated H_2_SO_4_. For plant biomass and yield, roots and shoots were weighed for fresh weight and then oven-dried at 70 °C to determine dry weight. Spike length and plant height were measured, and tillers were manually counted. Plants from each pot were harvested and weighed to estimate biomass yield. Panicles were manually separated, and grains were threshed, dried, and weighed to measure grain yield.

### Data analysis

The collected data were analyzed using one-way ANOVA in STATISTIX 8.1. Significant treatment means were compared using Tukey's Honestly Significant Difference (HSD) test at a 5% probability level.^53^ Figures were created using SigmaPlot 10, and trait relationships were analyzed using RStudio.

## Results

### Characterization of biochar

**Fig. 1:**
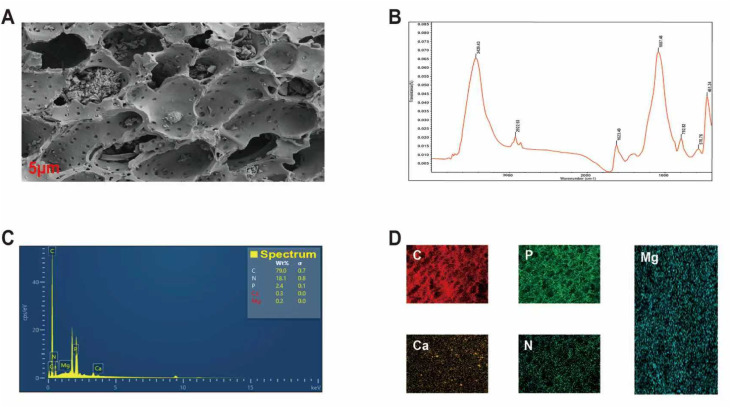
Morphological, chemical, and elemental characterization of biochar. Scanning electron microscopic (A), Fourier-transform infrared spectroscopy (B) and energy dispersive X-ray spectroscopy analysis (C and D).

Biochar was subjected to SEM, FTIR, and EDS analysis to determine its properties (Fig. 1). SEM analysis showed that the biochar had a porous structure, which is essential for immobilizing heavy metals. FTIR analysis revealed peaks at 3429.43, 2922.93, 1623.49, 1087.46, 792.82, and 461.34 cm^−1^, indicating the presence of O–H, C=O, C=C, C–O, and C–H functional groups, which help in immobilizing toxic metals. According to EDS analysis, the biochar contained 79% carbon, along with 18.1% nitrogen, 2.4% phosphorus, 0.3% calcium, and 0.2% magnesium (Fig. 1).

### Growth and yield parameters

The results showed that Cr and salinity caused a marked reduction in the growth traits of barley plants, with the most severe impacts observed under combined Cr + SS stress. Under combined Cr and salinity stress, root fresh weight (RFW), root dry weight (RDW), shoot fresh weight (SFW), and shoot dry weight (SDW) decreased by 7.88% (±0.086), 12.5% (±0.025), 11.92% (±0.238), and 6.85% (±0.0198), respectively, compared to Cr stress alone (Table 1). Biochar and silicon significantly mitigated the combined effects of Cr and SS and improved growth traits under Cr, SS, and co-exposure conditions (Table 1). Notably, the combination of BC and Si produced the best results, increasing RFW, RDW, SFW, and SDW by 30.70%, 47.22%, 50.03%, and 27.94%, respectively, under combined Cr + SS conditions (Table 1). Salinity and chromium stress also significantly reduced plant height and yield attributes compared to control conditions, with the greatest reduction observed under combined Cr + SS treatment (Table 1). Under Cr and SS, all amendments significantly enhanced the yield traits of barley. Co-applied BC and Si significantly increased plant height (PH), spike length (SL), 100-grain weight (100 GW), biomass yield (BY), and grain yield (GY) by 25.92% (±2.16), 34.50% (±0.246), 51.90% (±0.066), 42.38% (±0.372), and 50.84% (±0.523), respectively, under combined Cr + SS conditions (Table 1).

**Table 1:** Effect of biochar and silicon on growth and yield traits of barley under chromium and salinity stress^a^

Treatments	RFW (g)	RDW (g)	SFW (g)	SDW (g)	PH (cm)	SL (cm)	100 GW (g)	By (g)	GY (g)
CK	6.91 ± 0.13a	1.43 ± 0.11a	33.61 ± 1.24a	10.61 ± 0.37a	90.00 ± 1.63a	21.48 ± 0.76a	4.24 ± 0.13a	33.67 ± 0.97a	18.49 ± 0.45a
Cr	3.83 ± 0.11ij	0.79 ± 0.05ef	16.24 ± 0.21gh	6.08 ± 0.07gh	56.00 ± 3.74gh	11.62 ± 0.30f	2.21 ± 0.07h	14.49 ± 0.53h	10.03 ± 0.18g
SS	3.99 ± 0.05hi	0.82 ± 0.04ef	17.97 ± 0.30fg	6.34 ± 0.18gh	60.00 ± 1.63fgh	12.11 ± 0.18f	2.77 ± 0.09g	15.78 ± 0.63gh	11.92 ± 0.54f
Cr + SS	3.55 ± 0.08j	0.72 ± 0.02f	14.51 ± 0.24h	5.69 ± 0.20h	54.00 ± 3.55h	11.13 ± 0.22f	2.10 ± 0.07h	13.92 ± 0.54h	9.48 ± 0.18g
Cr + Si	4.90 ± 0.07de	1.14 ± 0.08	24.52 ± 1.57d	7.88 ± 0.14de	72.00 ± 1.64de	15.44 ± 0.44de	3.39 ± 0.11de	21.21 ± 0.81de	15.56 ± 0.47cd
Cr + BC	5.11 ± 0.08c	1.15 ± 0.08bc	26.12 ± 0.42cd	8.25 ± 0.14cd	74.00 ± 2.16cd	16.18 ± 0.45cd	3.63 ± 0.09cd	23.34 ± 0.85cd	16.22 ± 0.24bc
SS + Si	5.63 ± 0.10c	1.22 ± 0.05abc	27.56 ± 0.72bc	8.74 ± 0.22c	81.00 ± 0.82bc	17.64 ± 0.66c	3.90 ± 0.07bc	25.48 ± 0.88b	17.27 ± 0.22ab
SS + BC	6.17 ± 0.09b	1.37 ± 0.60ab	29.26 ± 0.78b	9.44 ± 0.18b	86.00 ± 2.82ab	19.75 ± 0.26b	4.08 ± 0.07ab	30.69 ± 0.45b	18.16 ± 0.27a
Cr + SS + BC	4.40 ± 0.12fg	0.90 ± 0.07def	20.68 ± 0.33ef	7.07 ± 0.05f	64.00 ± 2.20efg	14.58 ± 0.32e	3.14 ± 0.06fg	17.75 ± 0.61fg	14.00 ± 0.16ef
Cr + SS + Si	4.24 ± 0.04gh	0.99 ± 0.05cde	20.50 ± 0.22e	6.71 ± 0.15fg	63.00 ± 2.22fg	14.20 ± 0.25e	3.07 ± 0.04ef	16.15 ± 0.37gh	12.86 ± 0.52ef
Cr + SS + BC + Si	4.64 ± 0.10ef	1.06 ± 0.04cd	21.77 ± 0.40e	7.28 ± 0.11ef	68.00 ± 0.82def	14.97 ± 0.05de	3.19 ± 0.05ef	19.82 ± 1.18ef	14.30 ± 0.37de

a The data are average value of three replicates (*n* = 3) with ± SD and different letters within each column that shows significant differences at *p* < 0.05 based on HSD test. RFW: root fresh weight, RDW: root dry weight, SFW: shoot fresh weight, SDW: shoot dry weight, PH: plant height, SL: spike length, GW: grain weight, BY: biological yield, GY: grain yield.

### Photosynthetic pigments, leaf water contents and oxidative markers

**Fig. 2:**
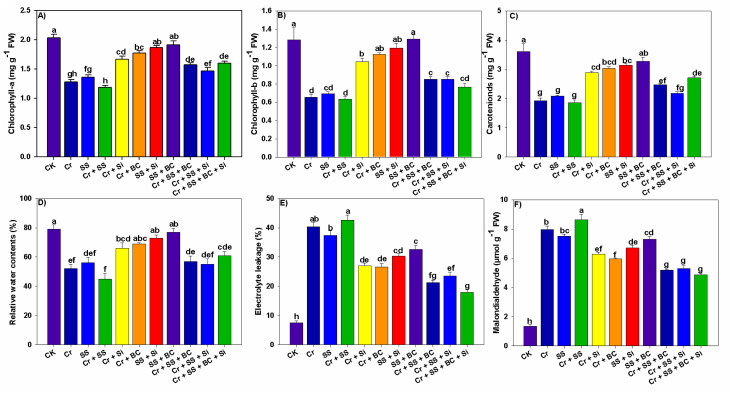
Effects of biochar and silicon on photosynthetic pigments (A–C), leaf water content (D), electrolyte leakage (E), and malondialdehyde (MDA) (F) in barley under chromium and salinity stress. Data are mean ± SD (*n* = 3). Different letters indicate significant differences at *p* < 0.05 (HSD test). CK: control, Cr: chromium, SS: salinity, BC: biochar, Si: silicon.

The results demonstrated that Chl-a, Chl-b, carotenoid content, and RWC were considerably decreased under SS, Cr, and Cr + SS compared to the control. However, BC and Si significantly enhanced Chl-a, Chl-b, and carotenoid content under SS and Cr stress conditions. Co-applying BC + Si significantly increased Chl-a, Chl-b, carotenoid content, and RWC by 35.59%, 22.22%, 46.23%, and 35.55%, respectively, under co-exposure to Cr + SS (Fig. 1). Electrolyte leakage was substantially increased in response to Cr, SS, and their combination compared to non-stressed plants (Fig. 2). Biochar and Si significantly reduced EL, with the most prominent decrease observed with co-applied BC + Si (Fig. 2). MDA production also increased under both stresses; however, the maximum increase was observed under combined Cr + SS (Fig. 2). All amendments significantly decreased MDA production in barley leaves, but the combination of BC and Si substantially reduced MDA by 77.57% (±0.108) under combined Cr and SS (Fig. 2). The results also showed that H_2_O_2_ levels were significantly increased under Cr, SS, and Cr + SS (Fig. 2). Biochar in combination with Si had positive effects and substantially decreased H_2_O_2_ by 72.40% (±0.114) under combined Cr and SS.

### Osmolytes and hormones

**Fig. 3:**
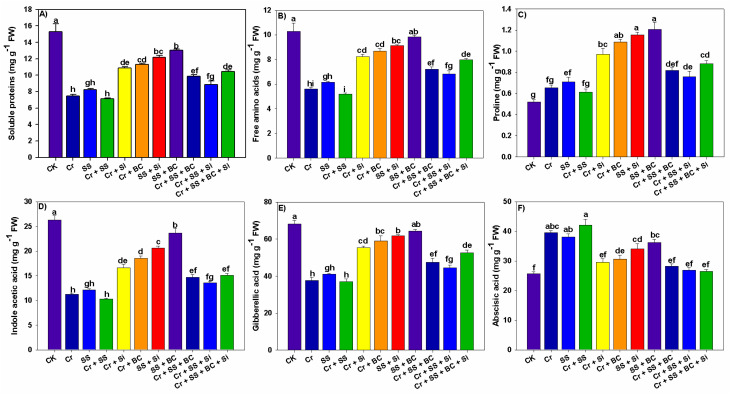
Effects of biochar and silicon on osmolytes (A–C) and phytohormones (D–F) in barley under chromium and salinity stress. Data are mean ± SD (*n* = 3). Different letters indicate significant differences at *p* < 0.05 (HSD test). CK: control, Cr: chromium, SS: salinity, BC: biochar, Si: silicon. CK: control, Cr: chromium, SS: salinity stress, BC: biochar, Si: silicon.

**Fig. 4:**
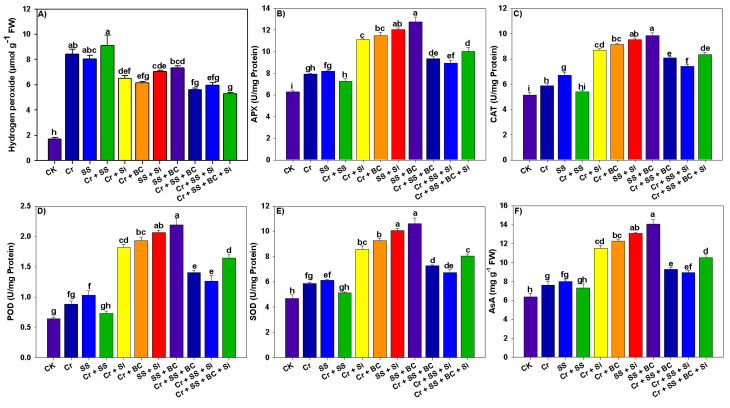
Effect of biochar and silicon application on hydrogen peroxide (A), antioxidants activities (B–F) of barley grown in chromium and salinity stress. Data are mean ± SD (*n* = 3). Different letters indicate significant differences at *p* < 0.05 (HSD test). CK: control, Cr: chromium, SS: salinity, BC: biochar, Si: silicon. CK: control, Cr: chromium, SS: salinity stress, BC: biochar, Si: silicon. APX: ascorbate peroxidase, CAT: catalase, POD: peroxidase, SOD: superoxide dismutase: AsA: ascorbic acid.

The results demonstrated that both stresses markedly decreased SP and FAA synthesis in barley plants (Fig. 3). Biochar and Si alleviated the Cr- and SS-mediated reduction in SP and FAA contents (Fig. 3). Notably, combining BC with Si showed promising results, and this combination considerably enhanced leaf SP and FAA contents by 45.87% and 54.05%, respectively (Fig. 3). Proline showed a contrasting response compared to SP and FAA, as its concentration increased in response to SS and Cr stress. Furthermore, BC and Si also enhanced proline synthesis under Cr + SS, helping to counteract stress conditions (Fig. 3). A contrasting response of hormones was observed under Cr, SS, and their combination. The synthesis of IAA (9.32%) and GA (2.13%) was significantly decreased under Cr + SS, while the synthesis of ABA (6.32%) was increased compared to Cr stress alone (Fig. 3). Notably, BC and Si effectively enhanced IAA and GA synthesis and decreased ABA synthesis, thus helping to maintain hormonal balance (Fig. 3). Biochar + Si increased IAA and GA contents by 46.64% and 42.17%, respectively, while decreasing ABA synthesis by 58.28% under Cr and SS treatment (Fig. 3). The activities of POD, CAT, SOD, AsA, and APX increased in response to both stresses, demonstrating that plants activated their defense systems to counteract oxidative damage (Fig. 4). Biochar and Si substantially increased antioxidant activities; however, a greater increase in the activity of all antioxidants such as APX (37.91%), CAT (54.73%), POD (124%), SOD (57.22%), and AsA (43.51%) was observed under combined Cr + SS with co-applied BC and Si (Fig. 4).

### Chromium and nutrient accumulation in barley plant

Chromium accumulation in different plant tissues (root, leaf, and grain) showed massive elevations under Cr alone and combined Cr + SS treatments. The maximum accumulation of Cr in roots, leaves, and grain was observed under combined Cr + SS treatment (Table 2), indicating that SS increased Cr uptake by plants. Biochar and Si effectively reduced Cr accumulation in roots, leaves, and grains by 44.21%, 55.50%, and 90%, respectively, compared to plants receiving no BC or Si (Table 2). The results also showed that Cr and SS significantly affected Na and Cl accumulation in barley plants. Notably, the maximum Na and Cl accumulation was observed under combined Cr and SS, indicating that the presence of Cr favored increased Na and Cl accumulation in barley plants (Table 3). Biochar and Si offset Na and Cl accumulation, with the maximum reduction of 89.22% and 51.20% under combined Cr + SS recorded with co-applied BC + Si (Table 3). Furthermore, Cr and SS caused a marked decrease in N, P, K, Ca, and Mg accumulation in barley plants (Table 3). The concentrations of N, P, K, Ca, and Mg in barley plants decreased by 32.52%, 30.24%, 41.05%, 53.95%, and 44.08%, respectively, under Cr + SS compared to Cr stress alone (Table 3). Biochar and Si mitigated the Cr- and SS-induced reduction in nutrient accumulation, and the maximum increase of 29.83%, 27.62%, 45.51%, 36.61%, and 41% in N, P, K, Ca, and Mg accumulation, respectively, under Cr + SS were found with co-applied BC and Si (Table 3).

**Table 2:** Effect of biochar and silicon on sodium, chloride, chromium and nutrient accumulation in barley plants grown under chromium and salinity stress^a^

Treatments	Root Cr (mg kg^−1^ DW)	Leaf Cr (mg kg^−1^ DW)	Grain Cr (mg kg^−1^ DW)
CK	—	—	—
Cr	14.10 ± 0.10b	5.94 ± 0.21a	0.95 ± 0.050b
SS	—	—	—
Cr + SS	16.08 ± 0.46a	6.50 ± 0.35a	1.14 ± 0.114a
Cr + Si	9.41 ± 0.14d	3.67 ± 0.16de	0.44 ± 0.012de
Cr + BC	8.89 ± 0.27d	3.27 ± 0.13e	0.39 ± 0.045e
SS + Si	—	—	—
SS + BC	—	—	—
Cr + SS + BC	12.23 ± 0.27b	4.53 ± 0.20bc	0.65 ± 0.064c
Cr + SS + Si	13.85 ± 0.38b	5.07 ± 0.14b	0.75 ± 0.050c
Cr + SS + BC + Si	11.15 ± 0.82c	4.18 ± 0.06 cd	0.60 ± 0.039 cd

a The data are average value of three replicates (*n* = 3) with ± SD and different letters within each column that shows significant differences at *p* < 0.05 based on HSD test. Na: sodium, Cl: chloride, Cr: chromium, N: nitrogen, P: phosphorus, K: potassium, Ca: calcium, Mg: magnesium.

**Table 3:** Effect of biochar and silicon on sodium, chloride, chromium and nutrient accumulation in barley plants grown under chromium and salinity stress^a^

Treatments	Na	Cl	N	P	K	Ca	Mg
	mg kg^−1^						
CK	0.98 ± 0.049e	4.91 ± 0.066e	42.43 ± 1.61a	23.89 ± 1.26a	53.88 ± 2.49a	64.17 ± 1.38a	48.68 ± 1.64a
Cr	1.04 ± 0.041e	5.13 ± 0.074e	23.61 ± 1.77gh	13.69 ± 0.58h	32.91 ± 1.00g	32.60 ± 1.26gh	29.90 ± 0.47g
SS	19.85 ± 0.52ab	35.67 ± 0.047a	26.46 ± 0.67fgh	14.48 ± 0.29fgh	36.44 ± 0.83ef	35.75 ± 0.60fgh	32.63 ± 1.25fg
Cr + SS	23.19 ± 0.82a	38.04 ± 1.92a	22.79 ± 0.99h	12.85 ± 0.46gh	29.33 ± 0.77fg	31.93 ± 1.65h	28.87 ± 1.59g
Cr + Si	1.14 ± 0.037e	5.63 ± 0.27e	31.29 ± 0.86de	17.83 ± 0.22de	46.42 ± 1.40bc	50.19 ± 1.58d	43.08 ± 1.23bcd
Cr + BC	1.37 ± 0.099e	6.14 ± 0.47e	34.39 ± 1.46cd	18.81 ± 0.49cd	48.13 ± 2.08b	54.13 ± 1.30cd	44.87 ± 2.60abc
SS + Si	17.65 ± 0.41bc	31.58 ± 1.28b	38.05 ± 1.17bc	20.54 ± 0.46bc	49.77 ± 2.09ab	57.82 ± 1.72bc	45.40 ± 0.82abc
SS + BC	16.30 ± 0.69bc	28.40 ± 0.73c	40.33 ± 0.86ab	22.55 ± 1.25ab	51.52 ± 0.84ab	60.70 ± 1.28ab	47.40 ± 0.82ab
Cr + SS + BC	11.61 ± 0.28cd	24.33 ± 0.40d	27.90 ± 0.94ef	16.07 ± 0.37ef	40.69 ± 0.51de	39.63 ± 0.42ef	38.62 ± 1.20de
Cr + SS + Si	13.41 ± 0.59d	25.84 ± 0.65cd	27.52 ± 0.70efg	15.80 ± 0.26efg	40.03 ± 1.71de	36.82 ± 1.15fg	36.65 ± 1.22ef
Cr + SS + BC + Si	10.49 ± 0.31d	23.59 ± 0.34d	29.59 ± 0.50ef	16.40 ± 0.33ef	42.68 ± 1.26cd	43.62 ± 1.77e	40.71 ± 1.27cde

a The data are average value of three replicates (*n* = 3) with ± SD and different letters within each column that shows significant differences at *p* < 0.05 based on HSD test. Na: sodium, Cl: chloride, Cr: chromium, N: nitrogen, P: phosphorus, K: potassium, Ca: calcium, Mg: magnesium.

### Soil properties

Chromium and SS significantly affected soil properties (Table 4). The results showed that Cr and SS decreased soil pH, while BC and Si increased soil pH (Table 4). The presence of salts in the growing medium enhanced Cr availability. Both biochar and Si decreased Cr availability, with better results observed with combined BC + Si application (Table 4). The availability of soil nutrients such as N, P, and K was markedly decreased under combined Cr and SS (Table 4). Biochar and Si substantially enhanced soil N, P, and K availability, with maximum increases of 50%, 29.26%, and 18.64%, respectively, under combined Cr and SS was observed with co-applied BC and Si (Table 4). Soil organic carbon concentration was also decreased by both stresses; however, co-exposure to Cr and SS caused the greatest reduction in SOC availability (Table 4). BC and Si supplementation increased SOC availability under both stress conditions (Table 4). Notably, BC, Si, and BC + Si considerably enhanced SOC availability by 19.21% (±0.062), 12.68% (±0.021), and 23.57% (±0.163), respectively, under combined Cr and SS (Table 4).

**Table 4:** Effect of biochar and silicon on sodium, chloride, chromium and nutrient accumulation in barley plants grown under chromium and salinity stress^a^

Treatments	Soil Cr (mg kg^−1^)	Soil pH	TN (mg kg^−1^)	AP (mg kg^−1^)	AK (mg kg^−1^)	SOC (g kg^−1^)
CK	—	7.72 ± 0.041a	0.30 ± 0.017a	11.27 ± 0.22a	156 ± 1.88a	5.50 ± 0.041a
Cr	24.69 ± 0.61ab	7.19 ± 0.029f	0.16 ± 0.009 fg	8.04 ± 0.13e	123 ± 1.70f	4.26 ± 0.042 fg
SS	—	7.21 ± 0.037f	0.17 ± 0.014efg	8.15 ± 0.08e	131 ± 1.41e	4.40 ± 0.049f
Cr + SS	26.88 ± 1.71a	7.15 ± 0.0.37f	0.14 ± 0.005g	7.62 ± 0.11f	118 ± 2.62f	4.06 ± 0.047g
Cr + Si	22.13 ± 0.82bc	7.45 ± 0.025cde	0.23 ± 0.016bcd	10.06 ± 0.37b	143 ± 2.16cd	5.01 ± 0.052 cd
Cr + BC	20.81 ± 0.93cd	7.53 ± 0.024bcd	0.24 ± 0.012bc	10.22 ± 0.057b	148 ± 2.17bc	5.08 ± 0.050 cd
SS + Si	—	7.57 ± 0.058bc	0.25 ± 0.005 ab	10.41 ± 0.14b	152 ± 1.25ab	5.19 ± 0.050bc
SS + BC	—	7.63 ± 0.033 ab	0.29 ± 0.012a	10.99 ± 0.074a	155 ± 2.44a	5.35 ± 0.054 ab
Cr + SS + BC	16.26 ± 0.43e	7.44 ± 0.034de	0.20 ± 0.017c–f	9.09 ± 0.079d	137 ± 1.17de	4.84 ± 0.021de
Cr + SS + Si	18.15 ± 0.86de	7.42 ± 0.017de	0.19 ± 0.008def	8.72 ± 0.037d	133 ± 1.25e	4.65 ± 0.16e
Cr + SS + BC + Si	13.40 ± 0.93f	7.36 ± 0.033e	0.21 ± 0.008b–e	9.58 ± 0.094c	140 ± 1.25d	4.91 ± 0.062d

a The data are average value of three replicates (*n* = 3) with ± SD and different letters within each column that shows significant differences at *p* < 0.05 based on HSD test. Na: sodium, Cl: chloride, Cr: chromium, N: nitrogen, P: phosphorus, K: potassium, Ca: calcium, Mg: magnesium.

## Discussion

The results demonstrated that combined Cr and SS exerted inhibitory effects on barley growth, as reflected by significant reductions in growth and biomass production (Table 1). The magnitude of growth reduction under combined stress was greater than that observed under individual stress conditions. Chromium toxicity interferes with root metabolic activities, disrupts nutrient uptake processes, and inhibits enzymatic functions essential for cell division and elongation.^2^ Concurrently, SS imposes osmotic stress and ionic imbalance, resulting in reduced water uptake, impaired turgor maintenance, and restricted cell expansion. Together, these stresses severely limit plant growth and structural development.^37,53,54^ We also observed that Cr and SS caused a considerable increase in Cr and Na accumulation within barley plants, which disrupted plant physiological functioning and hormonal balance, contributing to decreased plant growth (Table 1). Interestingly, SS and Cr also induced water deficiency, as indicated by a decrease in RWC. This reduction in water availability reduced nutrient uptake and accumulation and negatively affected plant functioning, thus leading to reduced growth and biomass (Table 1). However, to prevent water loss, barley plants reduced stomatal conductance to inhibit transpiration. This was confirmed by increased ABA synthesis under combined Cr and SS, which induced stomatal closure, aligning with previous studies.^55,56^ Photo-assimilation involves the conversion of light energy into chemical energy, a process in which chlorophyll plays a crucial role.^57^ In the present study, both stresses, particularly combined exposure, significantly decreased chlorophyll synthesis. Therefore, reduced chlorophyll synthesis decreases photo-assimilate production and leads to reduced growth and biomass.

Biochar and Si significantly mitigated the adverse effects of Cr and SS on barley plants. We observed that combined BC and Si produced more pronounced improvements in growth traits by increasing chlorophyll content, nutrient availability, antioxidant activities, and hormone synthesis, while decreasing Cr and Na availability. Biochar improves root growth and nutrient availability under stressful soil conditions.^58^ Moreover, biochar possesses a high surface area and functional groups capable of adsorbing heavy metals, thereby reducing Cr bioavailability and toxicity to plant roots.^59,60^ Silicon further enhances stress tolerance by strengthening cell walls, improving root architecture, and regulating ion uptake, particularly by limiting excessive sodium accumulation under saline conditions.^31,61^ The complementary actions of BC and Si collectively promoted growth recovery under combined stress. Reduced source strength and sink activity under stress conditions ultimately lead to poor grain filling and lower yield. The substantial improvement in yield attributes observed under BC + Si application reflects the cumulative positive effects of improved growth, enhanced photosynthetic efficiency, better water relations, and strengthened antioxidant defense (Fig. 4) and soil properties.^62^ By alleviating the physiological and biochemical constraints imposed by stress, BC + Si treatment enabled more efficient assimilate production and partitioning, resulting in improved yield performance even under adverse conditions.^63^

Combined Cr and SS caused a substantial decline in chlorophyll barley. These changes indicate severe damage to the photosynthetic apparatus. This decrease in chlorophyll content is attributed to reduced activity of enzymes involved in chlorophyll biosynthesis.^64^ Moreover, limitations in photosynthesis under combined SS and heavy metals occur through a distinct mechanism. For instance, stress conditions increase intercellular carbon dioxide, which arises from non-stomatal limitations.^65^ Furthermore, inhibited chlorophyll synthesis also inhibits light reactions and RuBisCO activity, thus leading to reduced photosynthesis.^66^ Biochar and Si, particularly in combination, significantly restored photosynthetic pigments. Biochar improves soil moisture retention and nutrient availability, ensuring a more favorable rhizosphere environment for water and nutrient uptake under saline conditions.^58^ Silicon contributes to the maintenance of photosynthetic efficiency by protecting chloroplast membranes, enhancing leaf water status, and regulating stomatal behavior under stress.^67^ The combined BC + Si treatment thus helped preserve chloroplast integrity, maintained water balance, and sustained photosynthetic activity, as reflected by improved pigment content.^62^ Notably, both BC and Si also enhanced Mg accumulation in barley plants grown under Cr and SS. This provides evidence that increased Mg accumulation contributed to increased chlorophyll synthesis, as Mg plays a structural role in chlorophyll. Biochar and Si also increased antioxidant activities, which in turn decreased oxidative damage and protected the photosynthetic apparatus, thus enhancing chlorophyll synthesis.

The results demonstrated that combined Cr and SS significantly decreased leaf RWC. This decrease was associated with poor root growth, which reduced water uptake and leaf RWC. Importantly, SS caused osmotic stress and induced water deficiency, which also contributed to decreased leaf RWC. In contrast, BC and Si maintained better RWC under stress conditions, indicating efficient water uptake by roots. Biochar and Si mitigated oxidative damage and maintained better root growth, thereby contributing to better water uptake and RWC. Exposure to combined Cr and SS resulted in a significant increase in H_2_O_2_ accumulation and EL, indicating excessive ROS production and compromised membrane integrity.^23^ Chromium and salinity disrupt cellular redox homeostasis by impairing electron transport chains and antioxidant systems, resulting in increased ROS production. The application of BC and Si markedly reduced oxidative stress markers and improved membrane stability (Fig. 2). Biochar reduced oxidative damage primarily by decreasing Cr availability in the soil, thereby limiting its uptake and subsequent ROS generation within plant tissues.^68^ Silicon further contributed to membrane stabilization by reinforcing cell walls, enhancing membrane integrity, and restricting the influx of toxic ions under saline conditions.^69^ The combined BC + Si treatment therefore, provided effective protection against oxidative injury by reducing ROS generation and enhancing membrane resistance to stress-induced damage.

The observed increase in the activities of antioxidant enzymes under stress conditions reflects an intrinsic adaptive response aimed at detoxifying excess ROS and maintaining cellular redox balance. However, this stress-induced activation of antioxidant enzymes was further amplified under BC + Si treatment, indicating a more robust and efficient antioxidant defense system. Silicon has been reported to enhance the expression and activity of antioxidant enzymes, thereby improving ROS scavenging capacity under stress conditions.^2^ Biochar indirectly supports antioxidant defense by improving nutrient availability, particularly of micronutrients required for enzyme synthesis and activation, and by creating a favorable soil environment.^70^ The accumulation of osmoregulatory compounds plays a critical role in stress tolerance. In the present study, Cr and SS decreased the accumulation of SP and FAA while increased proline accumulation. The decrease in SP and FAA was linked to increased oxidative damage and reduced N uptake and availability, which play critical roles in SP and FAA production. Proline is an important compatible compound that plays a crucial role in scavenging ROS and maintaining osmotic balance.^71^ The pronounced increase in proline accumulation under stress indicates a key mechanism for alleviating osmotic imbalance in barley plants. In addition to enzymatic antioxidants, the increased accumulation of SP and FAA under BC + Si treatment suggests improved osmotic adjustment and metabolic flexibility. These compounds contribute to cellular osmoprotection, stabilization of proteins and membranes, and maintenance of metabolic processes under stress conditions.^9^ Biochar and Si alleviated oxidative damage and enhanced nutrient availability, particularly N, which facilitated better SP and FAA synthesis under stress conditions.

Chromium and SS also disturbed hormonal balance, as evidenced by increased ABA and decreased IAA and GA accumulation. Increased ABA accumulation under stress conditions causes leaf senescence, impairs photosynthesis, and reduces plant growth.^71–73^ We observed that under Cr and Cr + SS conditions, BC and Si effectively reduced ABA accumulation. This decrease in ABA accumulation indicates that BC + Si effectively mitigates the adverse impacts of Cr and SS, thereby inducing a reduction in ABA accumulation.

Chromium accumulation in barley showing following order: roots > leaves > grains, indicating that most absorbed Cr was retained in root tissues, thereby limiting its translocation to aerial and edible parts. This aligns with earlier studies reporting that roots serve as a barrier to toxic metals, and metal sequestration in roots is a common strategy used by plants to reduce damaging impacts.^74,75^ However, the accumulation pattern is largely affected by the interaction between metals and SS under combined stress. Notably, competition between metal ions and Na^+^ can decrease or increase metal uptake. In our study, SS enhanced the uptake and accumulation of Cr, aligning with previous studies reporting that salinity affects heavy metal uptake, although this depends on the SS level and plant species.^76^ Sodium and Cl accumulation in barley increased under stress conditions, which was linked to increased availability of both ions. Biochar application significantly reduced Cr uptake by immobilizing the metal in the soil matrix, thereby decreasing its bioavailability to plant roots. Biochar contained a significant amount of carbon (Fig. 1) and functional groups (Fig. 1), which helped effectively immobilize Cr, thus decreasing its uptake and accumulation.^77^ Silicon further restricted root-to-shoot translocation of metals by strengthening root barriers.^78^ As a result, Cr concentration in aerial and above-ground parts was markedly reduced under BC + Si treatment, highlighting the potential of these amendments to improve food safety under Cr-contaminated and saline soil conditions.^79^ Biochar and Si also decreased Na accumulation, which was associated with the ability of BC to absorb Na^+^ ions on its surface, thus reducing their availability to plants.^80^

Nutrient accumulation was curbed under combined Cr and SS stress owing to intense competition between nutrients, Cr, and Na at uptake sites. Biochar effectively enhanced nutrient competition by increasing soil nutrient availability (Table 4) and possibly by decreasing the availability of Cr and SS (Table 3). The results demonstrated that BC and Si significantly decreased soil Cr availability. Biochar comprised 65% carbon; therefore, its application enhanced soil SOC. The dissolved organic matter released from BC contains hydroxyl and carboxyl groups, which chelate metal ions, thus decreasing their availability.^81^ We also observed that BC and Si increased soil pH, which caused Cr immobilization, hence reducing its availability. Biochar and Si effectively enhanced soil nutrient availability. Biochar and Si favor soil structure, nutrient retention, soil porosity, and aeration, and reduce competition between nutrients and heavy metals; therefore, they increase nutrient availability under combined metal and SS conditions.^82,83^

## Conclusion

Combined chromium and salinity severely inhibited growth by disrupting plant physiology, increasing oxidative damage, and promoting chromium and sodium accumulation. Confirming our hypothesis, biochar + silicon was superior to individual applications, achieving greater improvements in growth, yield, chlorophyll, antioxidant activity, and hormone synthesis, as well as greater reductions in oxidative damage and chromium and sodium uptake. The synergistic efficacy was attributed to: (i) biochar immobilizing chromium and improving soil properties, and (ii) silicon strengthening structural integrity, regulating ionic balance, and enhancing antioxidant defenses. This indicates biochar + silicon application is a cost-effective, sustainable strategy for improving barley productivity in salinity- and chromium-contaminated soils. Future studies should focus on long-term field evaluations, optimization of application rates, and assessment of microbial and molecular responses to further validate the potential of this integrated approach under diverse agro-ecological conditions.

## Author contributions

Imran Khan: supervision and writing – original draft; Aqsa Andleeb Sial: reviewing and editing, Shazia Hanif: reviewing and editing, Muhammad Nawaz: reviewing and editing, Muhammad Umer Chattha: supervisions and writing – original draft; Ayesha Mustafa: reviewing and editing, Muhammad Bilal Chattha: supervision and writing – original draft, Leeza Mujahid: reviewing and editing, Yasser S. Mostafa: reviewing and editing, Saad A. Alamri: reviewing and editing.

## Conflicts of interest

There are no conflicts to declare.

## Data Availability

The data supporting the conclusions of this article are included within the article.
